# Prediction of long-term adherence to direct oral anti-coagulants in patients with atrial fibrillation using first-order Markov models

**DOI:** 10.3389/fphar.2025.1673919

**Published:** 2025-10-16

**Authors:** Elias Edward Tannous, Shlomo Selitzky, Shlomo Vinker, Nicola Toukan, David Stepensky, Eyal Schwarzberg

**Affiliations:** ^1^ Department of Clinical Biochemistry and Pharmacology, Faculty of Health Sciences, Ben-Gurion University of the Negev, Beer-Sheva, Israel; ^2^ Pharmacy Services, Hillel Yaffe Medical Centre, Hadera, Israel; ^3^ Leumit Healthcare Services, Tel Aviv, Israel; ^4^ Department of Cardiology, Hillel Yaffe Medical Center, Hadera, Israel

**Keywords:** Markov, direct oral anti-coagulants, adherence, prediction, atrial fibrillation

## Abstract

Direct Oral Anti-Coagulants (DOACs) are the primary treatment for the long-term prevention of stroke in patients with atrial fibrillation. Strict adherence to DOAC therapy is crucial and must be maintained over the long term. Therefore, predicting long-term adherence is valuable for identifying patients at risk of non-adherence. We developed a novel method for predicting long-term adherence using first-order Markov models to assess adherence in new DOAC users during years 2–5. The prediction utilized age, CHA2DS2-VASc score, and first-year adherence data as predictors. Adherence was measured by calculating the proportion of days covered within consecutive 90-day windows, which were then stratified into deciles. We subsequently calculated the probability of a patient being in a specific adherence decile. The developed model demonstrated good calibration. We discovered that missing even 1 day of treatment per month in the first year was predictive of a lower likelihood of achieving the highest adherence decile in years 2–5. Additionally, we noted a non-linear relationship between age and adherence; adherence increased linearly with age but plateaued around age 75. This innovative approach to modelling and predicting adherence to DOACs for long-term therapy can help identify patients at risk of low adherence and may be applicable to other chronic medications.

## Introduction

Atrial fibrillation (AF) is the most commonly treated cardiac arrhythmia (Lip et al., 2012). Direct oral anticoagulants (DOACs) are widely used for the prevention of ischemic stroke in patients with AF. DOAC therapy is usually a lifelong therapy, and the extent of non-adherence was shown to be associated with worsened clinical outcomes (Borne et al., 2017; Ozaki et al., 2020). A large meta-analysis has found that up to 30% of AF patients are non-adherent to DOAC therapy (Salmasi et al., 2020).

Summary measures of adherence, such as 1-year proportion of days covered (PDC), do not capture adherence trajectory and may hide the patterns of long-term adherence (Hernandez et al., 2019; Salmasi et al., 2021). Therefore, group-based trajectory modeling (GBTM) has been suggested as an alternative (Hernandez et al., 2019; Alhazami et al., 2020; Chen et al., 2020; Salmasi et al., 2021). In several publications using GBTM, PDC was calculated for 90-day consecutive windows, spanning periods ranging from 3 to 5 years. Subsequently, patterns or groups were identified (Hernandez et al., 2019; Salmasi et al., 2021). While this method is an improvement over a single yearly PDC, it ultimately results in three to four groups that are modeled using multinomial regression. One extensive study has shown that while GBTM may identify interesting adherence groups, the ability to predict to which group a patient will be assigned was somewhat limited, reducing the clinical applicability of such models (Salmasi et al., 2021). Therefore, while this method may identify general patterns of adherence, it has little utility in the case of predicting adherence for a single patient.

Other methods to deal with longitudinal adherence data are available (Pinheiro and Bates, 2000). One such method is first-order Markov models (Rohde et al., 2024). In such models, also called transition models, the previous outcomes for the specific subject are included along with the covariates (Rohde et al., 2024). Applied to long-term adherence, the PDC of each 90-day window can be calculated for consecutive 90-day windows over 5 years. The PDC can be transformed to an ordinal scale, and then from the transition model, the probability of transition between adherence states can be obtained. A more relevant derived quantitative parameter is the probability of state occupation (Rohde et al., 2024). It can be derived from a first-order Markov model for each 90-day window. Such quantitative parameter is beneficial for clinicians as it allows them to calculate and visually present the probability of their patient’s PDC states for all the 5-year period.

Recently, we have shown that without data on the first months of adherence, PDC prediction is poor with baseline clinical, demographic, and socioeconomic predictors (Tannous et al., 2025). Based on these conclusions, the current study focuses on development of a first-order Markov model to predict 5-year adherence of DOACs in AF patients using the first-year adherence data as a predictor along with other covariates.

## Materials and methods

### Data collection

We collected social and demographic information from the electronic medical records. The electronic medical records of Leumit Healthcare Services (LHCS), one of Israel’s providers of public and semi-private health services, include data from multiple sources, including records of primary care physicians, community specialty clinics, hospitalizations, laboratories, and pharmacies. Diagnoses were captured in the registry through diagnosis-specific algorithms, utilizing the International Classification of Diseases, Ninth Revision (ICD-9) code reading and laboratory test results. The study protocol was approved by the LHCS Ethics Committee (Protocol number: 0022–22-LEU).

### Patient cohort

Candidate patients were screened from the LHCS database between January 2012 and December 2018. Patients under the age of 18 and pregnant women were excluded. Moreover, patients who died less than 5 years post-index date and patients who switched health insurance provider during the first 5 years post-index date were excluded.

Using a validated algorithm (Navar-Boggan et al., 2015), we included individuals if they had ≥3 recorded visits in the Medical Services Plan related to AF or atrial flutter (ICD-9 codes, 427427.3427.31427.32) with at least one of the recorded visits being AF-specific (ICD-9: 427.31, 427.32). At least two of the visits had to occur within 365 days (Navar-Boggan et al., 2015; Salmasi et al., 2021). Individuals with indications for oral anti-coagulants other than nonvalvular AF were excluded. Only patients who had prescriptions for DOAC for at least 2 months were included. Calculation of the minimal required sample size is available in the Supplementary Material.

### PDC calculation

PDC calculation was conducted according to the recommendations of a recently published scoping review and the TEN-SPIDERS tool (Dalli et al., 2022).

PDC was calculated for 90-day consecutive windows. In all cases, the denominator was 90. The numerator was calculated using the following method: First, adjustment of dates and identification of gaps post-index date was performed using the ‘adheRenceRX’ package in R, so that carry-over was granted for early refills of the same drug (Beal, 2020). Switching between different DOACs was allowed for, and the final PDC was calculated for DOACs as a group (i.e., carry-over was granted). Hospitalization days during the follow-up period were added to the numerator, and in-hospital supply was assumed since patients in hospitals in Israel do not use their own medications, as hospitals are required to supply them, unless contraindicated. The final formula used to calculate PDC was:

$$PDC=\frac{Gap\;adjusted\;covered\;days+\text{Inhospital}\;\text{days}}{90}$$

For the full TEN-SPIDERS tool, please see Supplementary Table S1 (Supplementary Material).

Subsequently PDC was transformed to an ordinal scale according to Table 1, to allow the use of first-order proportional odds Markov transition model.

**TABLE 1:** PDC states and their corresponding ranges.

PDC	PDC state
0%–9%	1
10%–19%	2
20%–29%	3
30%–39%	4
40%–49%	5
50%–59%	6
60%–69%	7
70%–79%	8
80%–89%	9
90%–100%	10

### First-order proportional odds Markov transition model

First-order proportional odds Markov transition models were used to analyze ordinal longitudinal data. A first-order Markov process models transitions from one period to the next, conditioning on the outcome (or state) at the previous period (hence the “first-order”) in addition to baseline covariates or predictors. After model fitting, a recursive matrix multiplication was used to perform unconditioning on previous states, yielding state occupancy probabilities (SOPs). State occupancy probabilities are conditional only on the previous state at the first prediction period and baseline covariates (Rohde et al., 2024). In the current study, the outcome was modeled as an ordinal variable, the PDC state at each consecutive 90-day window. The first-order Markov model for each period was conditioned on the PDC state of the previous period and time, in addition to baseline covariates.

### Model predictors

Based on previous studies, baseline clinical and demographic factors by themselves are poor predictors of PDC (Rymer et al., 2023; Tannous et al., 2025). In contrast, the first 90-day adherence data improved the 1-year PDC prediction (Tannous et al., 2025). Since we aimed to predict 5-year adherence, we hypothesized that adherence data (i.e., the number of days covered in 90-day windows) from the first year would be necessary to predict PDC in years 2–5. Therefore, our basic model included the first four 90-day windows as predictors. Moreover, since in previous studies adherence was associated with increasing age and CHA_2_DS_2_-VASc score (Hernandez et al., 2019; Salmasi et al., 2021), we tested whether the addition of age and CHA_2_DS_2_-VASc score would improve PDC prediction in comparison to 1-year adherence data alone. The number of days covered, age, and CHA_2_DS_2_-VASc score were modeled using restricted cubic splines with four knots to allow for a non-linear relationship with PDC.

### Model comparison

The basic model was compared to a model with a basic model with interaction with time for the third and fourth window adherence data, a model with only the first two 90-day windows adherence data (i.e., data from the first half of the first year), a basic model in addition to age and CHA_2_DS_2_-VASc score, a basic model and age alone, and a basic model and CHA_2_DS_2_-VASc score alone. Models were compared based on the Akaike Information Criteria (AIC) and the likelihood ratio (LR) test.

### Model calibration and discrimination

The final model was evaluated for calibration and discriminative performance. For model calibration, we produced a subject-level calibration curve, time-stratified.

Calibration, and subject-level calibration curve smoothed with generalized additive models. For state transition probabilities calibration we produced plots for PDC states 90%–100%,80%–89% and 0%–9%. For discrimination, we analyzed SOP Distribution Width (length of Inter Quartile Range (IQR)) Over time. A wider SOP distribution across states, predictions that allocate substantial probability to different categories rather than clustering near a single state, provide greater separation between these state-specific probabilities. Wider range leads to better discrimination between states. We used the bootstrap, with 100 repetitions, for internal validation of SOP Distribution Width. Moreover, we calculated the median and IQR for the absolute difference between SOP-derived mean time in state and observed time in PDC state 90%–100%.

### Predictor effects

Partial effects plots were used to examine the effect of continuous predictors on adherence state.

### State occupancy probabilities (SOPs) and mean time in state at 90-day windows

From the final model, SOPs were calculated for each 90-day window for the period between the start of the second year and the end of the fifth year. SOPs are calculated using the ‘soprobMarkovOrdm’ function from the ‘rms’ package in R. mean time in state was calculated from SOPs.

All statistical analyses were performed in R using ‘rms’ package (Harrell, 2024).

## Results

The study included data from 2,829 patients (see Supplementary Figure S1 in the Supplementary Material) with a nearly even sex distribution (49% female, 51% male) and a median age of 78 years. Hypertension was the most prevalent diagnosis (75%), followed by type 2 diabetes (55%), and heart failure (37%). CHA_2_DS_2_-VASc scores mostly ranged from three to 5. Table 2 presents demographic and clinical characteristics of the patients.

**TABLE 2:** Demographic and clinical characteristics of the patients who were included in the study, n = 2,82**9**.

Characteristic	Number of patients (%) or median (IQR)
Sex	
Female	1,382 (49%)
Male	1,447 (51%)
Age	78 (73, 85)
Diagnosis	
Atrial Fibrillation	2,678 (95%)
Atrial Flutter	151 (5.3%)
Heart Failure	1,044 (37%)
Hypertension	2,109 (75%)
Type 2 Diabetes	1,545 (55%)
Myocardial Infarction	295 (10%)
Stroke	952 (34%)
PAD	368 (13%)
CHA_2_DS_2_-VASc score	
Median (IQR)	5 (4,6)
1	41 (1.4%)
2	211 (7.5%)
3	448 (16%)
4	644 (23%)
5	607 (21%)
6	474 (17%)
7	280 (9.9%)
8	112 (4.0%)
9	12 (0.4%)
Number of days covered	
0–90 days	90 (85, 90)
90–180 days	90 (76, 90)
180–270 days	89 (72, 90)
270–360 days	89 (70, 90)
0–360 days	345 (287,360)

Overall, the median PDC state was high, 90%–100%, in all 90-day windows from year two to year 5 (see Table 3). However, the interquartile range changed from PDC 70–79%-90%–100% in the first 90-day windows of year two to 0–9%-90%–100% in the last 90-day windows of year 5, indicating that overall there was a decline in PDC state over time. Table 3 presents PDC states across all 90-day windows from years two–5. About two-thirds of patients were prescribed Apixaban, followed by Rivaroxaban and dabigatran. 11.7% of patients switched between DOAC agents (Supplementary Table S2; Supplementary Material). PDC variation by DOAC agent is presented in table (Supplementary Table S3; Supplementary Material).

**TABLE 3:** PDC states over all 90-day windows from years two–5.

Period, days	Period, years	N = 2,829^a^
360–450 days PDC ordinal	1.0–1.25	90%–100% (70%–79%–90%–100%)
450–540 days PDC ordinal	1.25–1.50	90%–100% (70%–79%–90%–100%)
540–630 days PDC ordinal	1.50–1.75	90%–100% (60%–69%–90%–100%)
630–720 days PDC ordinal	1.75–2.0	90%–100% (70%–79%–90%–100%)
720–810 days PDC ordinal	2.0–2.25	90%–100% (60%–69%–90%–100%)
810–900 days PDC ordinal	2.25–2.5	90%–100% (60%–69%–90%–100%)
900–990 days PDC ordinal	2.5–2.75	90%–100% (60%–69%–90%–100%)
990–1,080 days PDC ordinal	2.75–3.0	90%–100% (60%–69%–90%–100%)
1,080–1,170 days PDC ordinal	3.0–3.25	90%–100% (50%–59%–90%–100%)
1,170–1,260 days PDC ordinal	3.25–3.5	90%–100% (50%–59%–90%–100%)
1,260–1,350 days PDC ordinal	3.5–3.75	90%–100% (40%–49%–90%–100%)
1,350–1,440 days PDC ordinal	3.75–4.0	90%–100% (20%–29%–90%–100%)
1,440–1,530 days PDC ordinal	4.0–4.25	90%–100% (20%–29%–90%–100%)
1,530–1,620 days PDC ordinal	4.25–4.5	90%–100% (10%–19%–90%–100%)
1,620–1,710 days PDC ordinal	4.5–4.75	80%–89% (10%–19%–80%–89%)
1,710–1,800 days PDC ordinal	4.75–5.0	90%–100% (0%–9%–90%–100%)
1,800–1,890 days PDC ordinal	5.0–5.25	90%–100% (0%–9%–90%–100%)

^*a*^ n (%); Median (IQR).

### Model choice and comparison

The Basic model plus age and CHA_2_DS_2_-VASc score, was compared to several other models using AIC and LR test (Table 4). The basic model plus age and CHA_2_DS_2_-VASc score with time interaction with the third and fourth window adherence had the lowest AIC and was used as the final model in all further analyses.

**TABLE 4:** Model comparison using Akaike Information Criterion (AIC) and Likelihood Ratio (LR) Test.

Model	AIC	P-value LR test
Age, CHA_2_DS_2_-VASc score, first 90-day window, second 90-day window, third 90-day window fourth 90-day window	86,775	-
Age, CHA_2_DS_2_-VASc score, first 90-day window, second 90-day window, third 90-day*time, window fourth 90-day window*time (interaction with time added for third and fourth window)	86,676	<2.2e-16
Age, CHA2DS2-Vasc score, first 90-day window, second 90-day window	87,783	<2.2e-16
CHA2DS2-Vasc score, first 90-day window, second 90-day window, third 90-day window fourth 90-day window	86,891	<2.2e-16
Age, first 90-day window, second 90-day window, third 90-day window fourth 90-day window	86,804	2.98e-08
first 90-day window, second 90-day window, third 90-day window fourth 90-day window	87,016	<2.2e-16
Age, CHA2DS2-Vasc score, first 90-day window, second 90-day window, third 90-day window	87,239	<2.2e-16
Age, CHA2DS2-Vasc score, first 90-day window, second 90-day window, fourth 90-day window	87,064	<2.2e-16
Age, CHA2DS2-Vasc score, second 90-day window, third 90-day window fourth 90-day window	86,777	0.0736
Age, CHA2DS2-Vasc score, first 90-day window, third 90-day window fourth 90-day window	86,792	4.38e-05

### Model calibration and discrimination

**FIGURE 1:**
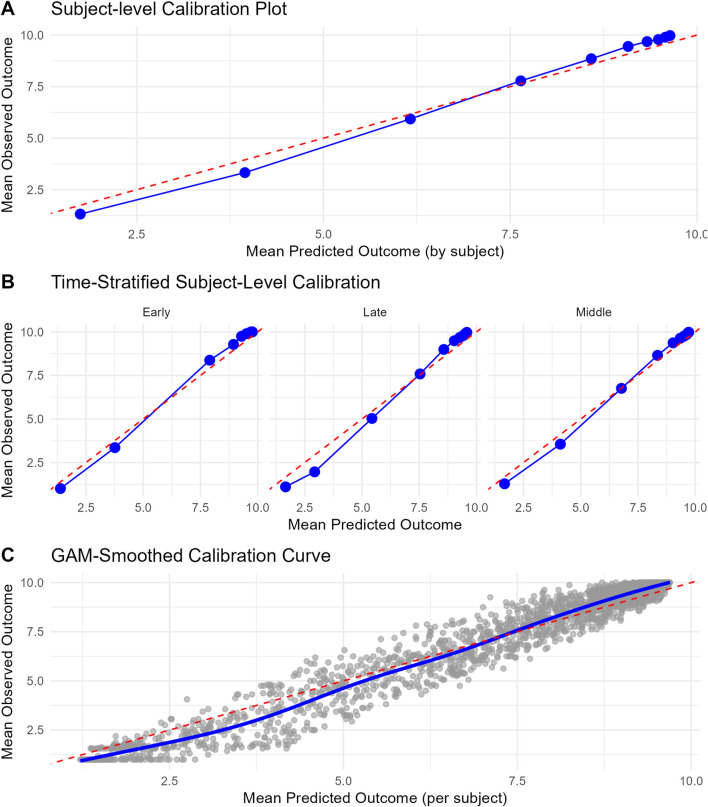
Calibration curves for the final model. **(A)** Subject-level calibration curve. Each point signifies a decile group of subjects, classified based on their mean predicted outcome. The x-axis shows the mean predicted outcome per decile, while the y-axis shows the corresponding mean observed outcome. The dashed red diagonal line denotes perfect calibration. **(B)** Time-stratified calibration curve. These curves illustrate the concordance between predicted and observed outcomes across various time periods (Early = first 90 days window in year 2, middle = from the second 90-day window of year two till year 3, and late = from year three to year 5). For each period, subjects were grouped into deciles according to their mean predicted outcome (expected value of predicted state probabilities), and the mean observed outcomes were computed within each group. Each panel presents a distinct time stratum, with points indicating decile averages and the dashed red line representing perfect calibration. **(C)** Subject-level calibration curve smoothed with generalized additive models (GAM). This curve depicts the relationship between mean predicted and observed outcomes at the individual subject level, with a smooth trend estimated *via* a GAM. Grey points denote individual subjects, and the blue curve illustrates the GAM-smoothed calibration line. The dashed red diagonal line signifies perfect calibration.

**FIGURE 2:**
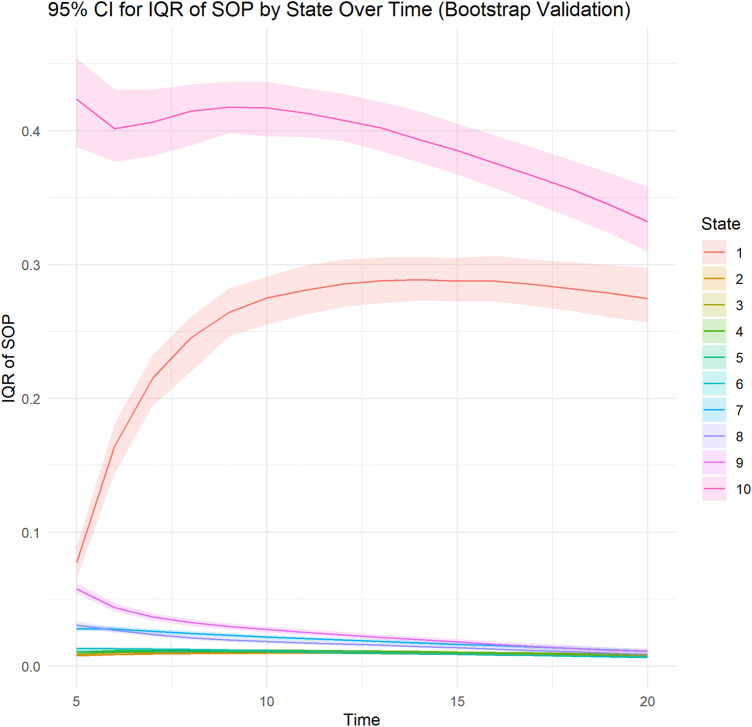
Bootstrapped Distribution of SOP Width (interquartile range length) for PDC states over time. A line plot illustrates the bootstrapped median of the interquartile range (IQR length) of predicted state occupancy probabilities (SOPs) across different time points for each ordinal state (ranging from PDC 0%–9% to PDC 90%–100%). The shaded area represents the bootstrapped upper (97.5 percentile) and lower (2.5 percentile) confidence limits. The IQR length measures the variability of predicted probabilities across subjects at each time point, with wider IQRs signifying greater discrimination capacity of the model in distinguishing subjects’ likelihood of occupying specific states. Each line corresponds to a distinct ordinal state.

Subject-level calibration curve, time stratified calibration curve, subject-level calibration curve smoothed with generalized additive models are presented in Figure 1. The model appears to be well calibrated. State transition probabilities calibration plots for PDC states 90%–100%, 80%–89% and 0%–9% are presented in Supplementary Figures S4–S6. SOP Distribution width varied between PDC states. SOP Distribution Width (length IQR) was highest for PDC state 90%–100% with an average of 0.398 across time and for PDC state 0%–9% with an average of 0.282 across time. These results indicate that for PDC state 90%–100% and PDC state 0%–9% the model shows relatively good discriminative ability. Bootstrapped internal validation of SOP Distribution Width (length IQR) was highest for PDC state 90%–100% with a median 0.391 (bootstrapped 95% CI 0.370–0.413) across time and for PDC state 0%–9% with a median of 0.255 (bootstrapped 95% CI 0.237–0.273) across time. These results indicate that for PDC state 90%–100% and PDC state 0%–9% the model shows relatively good discriminative ability. For all other PDC states SOP Distribution Width ranged between 0.009 and 0.027, indicating relatively weak discriminative ability for these states. Bootstrapped SOP Distribution Width (length of IQR) over time is presented in Figure 2.

Moreover, we calculated Median Absolute Error of mean time in the highest PDC state (90%–100%). Median Absolute Error was 2.95 (IQR 1.40–4.28).

Two additional sensitivity analyses were performed. In the first analysis we fitted the same final model but with four states instead of ten; 0%–9%, 10%–69%, 70%–89%, 90%–100% and in the second we fitted the same final model but with three states instead of ten; 0%–9%, 10%–89%, 90%–100% and evaluated the models discriminative ability. The results are presented in Supplementary Figures S2, S3 in the Supplementary Material. As in the original analysis, discrimination performance was good for the extreme states 0%–9% and 90%–100% but weak for the intermediate states regardless of the method of grouping.

### Predictor effects

**FIGURE 3:**
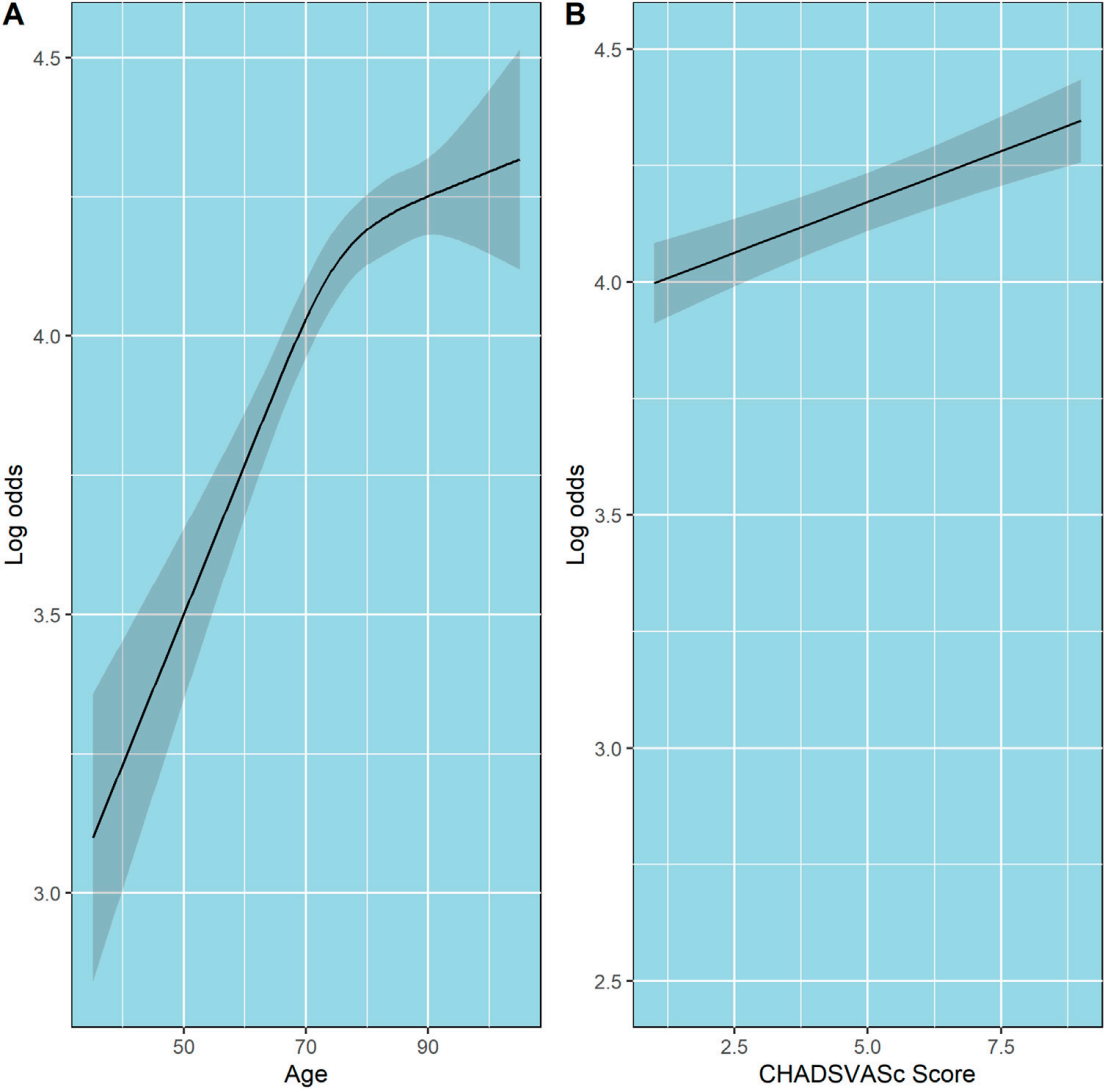
Partial effects of age and CHA_2_DS_2_-VASc score on log odds of ordinal PDC category for DOACs. Point estimates and confidence intervals for effects of individual predictors are computed holding other predictors to selected constants (mean for numeric variables). **(A)** Age. **(B)** CHA_2_DS_2_-VASc score. The shaded zones indicate the 95% confidence intervals.

**FIGURE 4:**
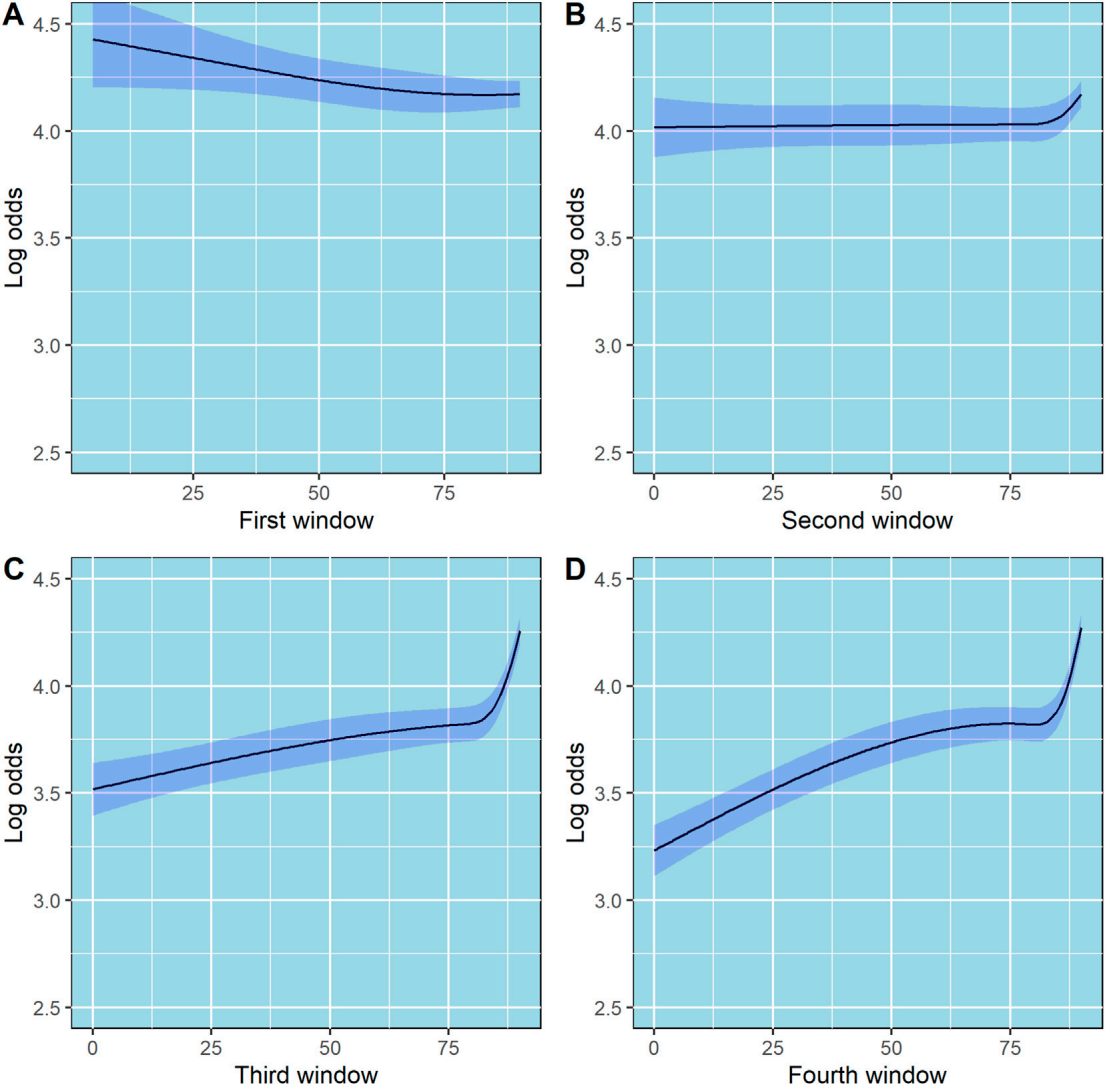
Partial effects of the first, second, third and fourth 90-day windows on log odds of ordinal PDC category for DOACs. Point estimates and confidence intervals for effects of individual predictors are computed holding other predictors to selected constants (mean for numeric variables). **(A)** First 90-day window. **(B)** Second 90-day window. **(C)** Third 90-day window. **(D)** Fourth 90-day window. The shaded zones indicate the 95% confidence intervals.

The partial effects of age, CHA_2_DS_2_-Vasc score, and the effect of the first four 90-day windows adherence data on log odds of PDC state are shown in Figures 3, 4, respectively. Additionally, since age is included in the calculation of CHA_2_DS_2_-Vasc score we investigated the possible correlation between age and CHA_2_DS_2_-Vasc score. The linear correlation between age and CHA_2_DS_2_-Vasc score was weak (p = 0.4) and the effect of the presence of CHA_2_DS_2_-Vasc score in the model on age was minimal (see Supplementary Figure S7 in the Supplementary Material).

### State occupancy probabilities at 90-day windows

**FIGURE 5:**
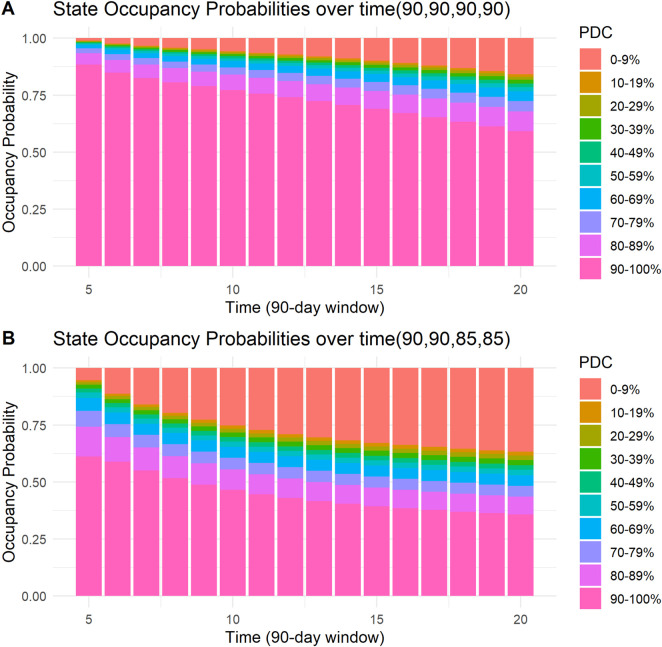
State occupancy probabilities. Y-axis shows state occupancy probabilities at consecutive 90-day windows, starting from the fifth 90-day window to the 20th 90-day window. (x-axis) State occupancy probabilities were calculated with age and CHA_2_DS_2_-VASc score held constant at 99 and 99, respectively. **(A)** With the first four windows having 90, 90, 90, and 90 days covered, respectively. **(B)** With the first four windows having 90, 90, 85 and 85 days covered, respectively.

**FIGURE 6:**
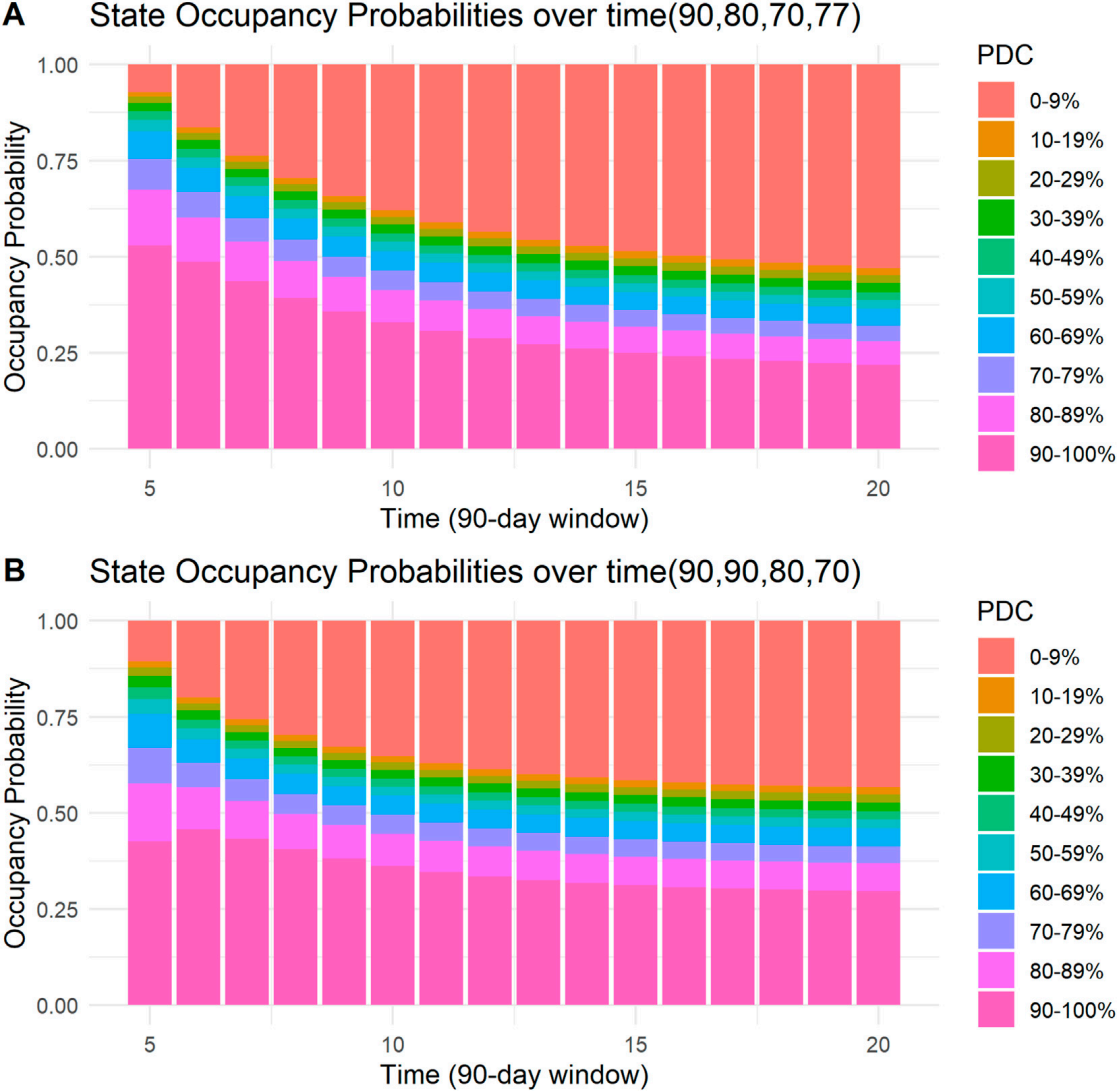
State occupancy probabilities. Y-axis shows state occupancy probabilities at consecutive 90-day windows, starting from the fifth 90-day window to the 20th 90-day window. (X-axis) state occupancy probabilities were calculated with age and CHA_2_DS_2_-VASc score held constant at 99 and 99, respectively. **(A)** With the first four windows having 90, 80, 70, and 77 days covered, respectively. **(B)** With the first four windows having 90,90, 80, and 70 days covered, respectively.

Four examples of the visual presentation of SOPs along the follow-up period are shown in Figures 5, 6. In Figure 5, the contrast between a patient with all days covered in the first four 90-day windows and a patient who misses only 5 days in the third and fourth windows is shown. Predicted mean time in PDC 90%–100% was 11.54 (90-day units) for a patient with all days covered in the first four 90-day windows, compared to a predicted mean time in PDC 90%–100% of 7.15 (90-day units) for a patient who missed only 5 days in the third and in the fourth 90-day windows. R code and code for a Shiny application to reproduce these figures and similar ones are provided in the Supplementary Material.

## Discussion

We developed a first-order Markov model to predict 5-year adherence of patients on DOACs. This novel approach enabled the derivation of PDC state occupancy probabilities for each 90-day window over 5 years. This method allows for the longitudinal modeling of PDC over extended periods. It provides a straightforward approach to visualize the probability of a patient on DOAC being in a specific PDC state. Our model demonstrated good discrimination performance for PDC states of 90%–100% and 0%–9%. For other PDC states, discrimination was weak. In addition, this approach allows for the estimation and comparison between patients of the expected mean time in a particular PDC state.

Moreover, we used the first-year adherence data (from the first four 90-day windows) as predictors of long-term adherence. Interestingly, using restricted cubic splines to model the first four 90-day windows showed an interesting relation between number of days covered and adherence (Figure 4). In the third and fourth 90-day windows, the slope is steep in the fourth knot, where days covered is equal to 89, suggesting that there is a significant increase in the odds of being at higher PDC state in patients whose number of days covered is 89 or 90, as compared to lower values. This finding implies that patients who miss 1 day of DOACs per month will have a dramatically lower probability of staying in the highest PDC state (PDC 90%–100%) through a 5-year period as compared to patients who miss none or 1 day per 3 months. Focusing, for example, on patients who miss 1–2 days of DOACs per month may significantly increase their probability of staying in the highest PDC state. Presumably, such patients are convinced of the necessity and efficacy of the treatment, otherwise they would not continue purchasing their medications for such a long period but may experience occasional forgetfulness. Therefore, strategies such as sending SMS massages to remind them to purchase their DOACs or occasional reminders by phone calls, may be helpful (Whitmore et al., 2025). Persistently adhering to extended DOAC therapy is linked to a lower risk of recurrent VTE without increasing major bleeding. Even small decreases in DOAC adherence among patients with atrial fibrillation are associated with significant increases in the risk of stroke (Park et al., 2023).

The relationship between age and the probability of being in a higher PDC state was also interesting and showed a non-linear pattern. At ages younger than 75, there is a linear increase in log-odds of being at a higher PDC state as age increases. However, at ages above 75, a quasi-plateau is reached. Borne *et al.* and Hernandez *et al* had found that increased age is associated with increased adherence; In contrast, we found that this relationship is not linear (Borne et al., 2017; Hernandez et al., 2019). However, this non-linear pattern could reflect the effect of increased comorbidities rather than age.

Although an increased CHA_2_DS_2_-VASc score was associated with an increased probability of being at a higher PDC state, the increase was modest. For example, compared with patients with CHA_2_DS_2_-VASc scores of 4, patients who had CHA_2_DS_2_-VASc scores of six had higher odds of being at a higher PDC state, OR = 1.091 (95% CI 1.057–1.125). These results are consistent with previous studies (Borne et al., 2017; Hernandez et al., 2019). This might be due to heightened awareness of consequences of nonadherence to DOACs in those with higher risk.

The adherence rates in the current study are consistent with those in previous studies. For example, a nationwide survey from Belgium reported a mean PDC of 97.3% in the first year compared to a median of 99.4% in the current study (Sabaté et al., 2021). Moreover, in the current study, patients on Dabigatran had a lower probability of having PDC >90% compared to patients on Apixaban or Rivaroxaban (54.7% vs. 59.8% and 61.2%, respectively). These results are consistent with a cross-national comparison in six European Countries, which found that patients on Dabigatran had the highest dropout rate (Grymonprez et al., 2022).

We proposed an approach for modeling long-term adherence. The other principal approach in the literature is GBTM. GBTM offers an improvement over calculating a single 5-year PDC, and is primarily used for population-level pattern identification (Hernandez et al., 2019; Salmasi et al., 2021; An et al., 2021; Mohan et al., 2022; Park et al., 2023; Fatima et al., 2024; Mohan et al., 2024) However, predicting to which trajectory a patient would belong is challenging and Salmasi et al. found that only a few variables were linked to any particular trajectory (Salmasi et al., 2021). Therefore, GBTM is less suitable for individual prediction. The approach we proposed could serve as a suitable method for estimating probabilities of PDC states for a specific patient for each 90-day window across 5 years.

### Limitations

The current study has several limitations. First, we assumed that patients who purchased DOACs administered them as prescribed, an assumption we cannot test. Second, we only included patients who had filled at least two prescriptions of DOACs; therefore, our results do not apply to patients who never initiate DOAC therapy. Third, predictions of adherence can only be generated after the first year of use; therefore, other tools should be used for adherence prediction during the first year. Fourth, for PDC states other than the highest and lowest states, discrimination was relatively weak. Fifth, we have not shown that model guided intervention improves patient outcomes. Finally, we did not perform model validation on a new dataset (i.e., external validation).

### Implications for practice

The approach to modeling longitudinal DOAC adherence has several implications for practice. First, patients with a relatively low probability of being at a PDC state between 90% and 100% can be identified and prioritized. Ruff et al., using a simulation pharmacokinetic-pharmacodynamic approach, suggested a threshold of PDC >90% to discriminate between patients with low or high risk of non-adherence (Ruff et al., 2019). Our approach enables clinicians and policy managers to identify such patients and implement interventions that enhance adherence. We provided the R code that enables the implementation of model-based prediction of PDC into decision support systems. Additionally, the individual clinician can use the Shiny applications to generate graphs of state occupancy probability predictions. Second, the finding that missing even 1–2 days a month in the first year of DOAC use significantly decreases the probability of maintaining PDC >0.9 in the long term can be used to identify patients at risk of lower long-term adherence probability and possible ill-health consqeuences. Therefore, while such patients may have been deemed adherent in the past, our findings suggest that they are nevertheless at risk of low adherence in the long term. Third, the relatively high discrimination of the model for the lowest PDC state (0%–9%) means that a clinician can identify which patients are at high risk of being at the lowest PDC state. Non-adherence causes recurrent cardio-embolic events and a higher risk of mortality, especially in the population with a high CHA2DS2-VASc score. In such patients, it is crucial to identify the risk of low PDC (0%–9%) promptly, and the current model allows that. Fourth, while the current study cannot identify patient groups who might benefit most from adherence-enhancing interventions, the method introduced—using a first-order Markov proportional odds model—can be used to examine how a specific intervention would affect PDC in the long term and how this effect might differ between patient subgroups.

## Conclusion

We developed a first-order Markov model for 5-year DOAC adherence data. This new approach to modeling long-term adherence allows the calculation of the probability to occupy PDC states in consecutive 90-day windows for 5 years. This tool enables the identification of patients with low probabilities of occupying the highest PDC state. Moreover, we found that even missing as little as one to 2 days a month in the first year of DOAC is predictive of a lower probability of staying at higher PDC states in the long term.

## Data Availability

The datasets presented in this article are available by request to the corresponding author and after approval from Leumit Healthcare Services. Requests to access the datasets should be directed to ET, tanus@bgu.ac.il.

## References

[B1] Alhazami M. Pontinha V. M. Patterson J. A. Holdford D. A. (2020). Medication adherence trajectories: a systematic literature review. JMCP 26 (9), 1138–1152. 10.18553/jmcp.2020.26.9.1138 32857646 PMC10391275

[B2] An J. Bider Z. Luong T. Q. Cheetham T. C. Lang D. T. Fischer H. (2021). Long-term medication adherence trajectories to direct oral anticoagulants and clinical outcomes in patients with atrial fibrillation. J. Am. Heart Assoc. 10 (21), e021601. 10.1161/JAHA.121.021601 34713708 PMC8751846

[B3] Beal B. _adheRenceRX: assess medication Adherence from pharmaceutical claims data_. R package 2020. Version 1.0.0. Available online at: https://CRAN.R-project.org/package=adheRenceRX.

[B4] Borne R. T. O’Donnell C. Turakhia M. P. Varosy P. D. Jackevicius C. A. Marzec L. N. (2017). Adherence and outcomes to direct oral anticoagulants among patients with atrial fibrillation: findings from the veterans health administration. BMC Cardiovasc. Disord. 17, 236–237. 10.1186/s12872-017-0671-6 28865440 PMC5581418

[B5] Chen N. Brooks M. M. Hernandez I. (2020). Latent classes of adherence to oral anticoagulation therapy among patients with a new diagnosis of atrial fibrillation. JAMA Netw. open 3 (2), e1921357. 10.1001/jamanetworkopen.2019.21357 32074287 PMC7081375

[B6] Dalli L. L. Kilkenny M. F. Arnet I. Sanfilippo F. M. Cummings D. M. Kapral M. K. (2022). Towards better reporting of the proportion of days covered method in cardiovascular medication adherence: a scoping review and new tool TEN-SPIDERS. Br. J. Clin. Pharmacol. 88 (10), 4427–4442. 10.1111/bcp.15391 35524398 PMC9546055

[B7] Fatima B. Mohan A. Altaie I. Abughosh S. (2024). Predictors of adherence to direct oral anticoagulants after cardiovascular or bleeding events in medicare advantage plan enrollees with atrial fibrillation. JMCP 30 (5), 408–419. 10.18553/jmcp.2024.30.5.408 38701026 PMC11068655

[B8] Grymonprez M. Capiau A. Steurbaut S. Mehuys E. Boussery K. De Backer T. L. (2022). Adherence and persistence to oral anticoagulants in patients with atrial fibrillation: a Belgian nationwide cohort study. Front. Cardiovasc. Med. 9, 994085–2022. 10.3389/fcvm.2022.994085 36247477 PMC9558210

[B9] Harrell Jr F. E. (2024). _rms: regression modeling strategies_. R package version 6.8-0. Available online at: https://CRAN.R-project.org/package=rms.

[B10] Hernandez I. He M. Chen N. Brooks M. M. Saba S. Gellad W. F. (2019). Trajectories of oral anticoagulation adherence among medicare beneficiaries newly diagnosed with atrial fibrillation. J. Am. Heart Assoc. 8 (12), e011427. 10.1161/JAHA.118.011427 31189392 PMC6645643

[B11] Lip G. Y. Brechin C. M. Lane D. A. (2012). The global burden of atrial fibrillation and stroke: a systematic review of the epidemiology of atrial fibrillation in regions outside North America and Europe. Chest 142 (6), 1489–1498. 10.1378/chest.11-2888 22459778

[B12] Mohan A. Majd Z. Trinh T. Paranjpe R. Abughosh S. M. (2022). Group based trajectory modeling to assess adherence to oral anticoagulants among atrial fibrillation patients with comorbidities: a retrospective study. Int. J. Clin. Pharm. 44 (4), 966–974. 10.1007/s11096-022-01417-4 35776377

[B13] Mohan A. Chen H. Deshmukh A. A. Wanat M. Essien E. J. Paranjpe R. (2024). Group-based trajectory modeling to identify adherence patterns for direct oral anticoagulants in medicare beneficiaries with atrial fibrillation: a real-world study on medication adherence. Int. J. Clin. Pharm. 46 (6), 1525–1535. 10.1007/s11096-024-01786-y 39190225

[B14] Navar-Boggan A. M. Rymer J. A. Piccini J. P. Shatila W. Ring L. Stafford J. A. (2015). Accuracy and validation of an automated electronic algorithm to identify patients with atrial fibrillation at risk for stroke. Am. Heart J. 169 (1), 39–44.e2. 10.1016/j.ahj.2014.09.014 25497246

[B15] Ozaki A. F. Choi A. S. Le Q. T. Ko D. T. Han J. K. Park S. S. (2020). Real-world adherence and persistence to direct oral anticoagulants in patients with atrial fibrillation: a systematic review and meta-analysis. Circ. Cardiovasc. Qual. Outcomes 13 (3), e005969. 10.1161/CIRCOUTCOMES.119.005969 32148102

[B16] Park H. Jones B. L. Huang P. Kang H. Dietrich E. A. DeRemer C. E. (2023). Trajectories of oral anticoagulation adherence and associated clinical outcomes during long-term anticoagulation among medicare beneficiaries with venous thromboembolism. Ann. Pharmacother. 57 (12), 1349–1360. 10.1177/10600280231155489 36999519

[B17] Pinheiro J. Bates D. (2000). Mixed-effects models in S and S-PLUS. Springer science and business media.

[B18] Rohde M. D. French B. Stewart T. G. Harrell Jr., F. E. (2024). Bayesian transition models for ordinal longitudinal outcomes. Stat. Med. 43 (18), 3539–3561. 10.1002/sim.10133 38853380

[B19] Ruff C. Koukalova L. Haefeli W. E. Meid A. D. (2019). The role of adherence thresholds for development and performance aspects of a prediction model for direct oral anticoagulation adherence. Front. Pharmacol., 10–2019. 10.3389/fphar.2019.00113 30837879 PMC6389873

[B20] Rymer J. A. Wegermann Z. K. Kaltenbach L. A. Webb L. E. Peterson E. D. Wang T. Y. (2023). Challenge in predicting persistence to P2Y12 inhibitors: a perspective from the ARTEMIS trial. J. Am. Heart Assoc. 12, e029063. 10.1161/JAHA.122.029063 37301758 PMC10356056

[B21] Sabaté M. Vidal X. Ballarin E. Rottenkolber M. Schmiedl S. Grave B. (2021). Adherence to direct oral anticoagulants in patients with non-valvular atrial fibrillation: a cross-national comparison in six European countries (2008–2015). Front. Pharmacol. olume, 12–2021. 10.3389/fphar.2021.682890 34803665 PMC8596153

[B22] Salmasi S. Loewen P. S. Tandun R. Andrade J. G. De Vera M. A. (2020). Adherence to oral anticoagulants among patients with atrial fibrillation: a systematic review and meta-analysis of observational studies. BMJ Open 10 (4), e034778. 10.1136/bmjopen-2019-034778 32273316 PMC7245382

[B23] Salmasi S. De Vera M. A. Safari A. Lynd L. D. Koehoorn M. Barry A. R. (2021). Longitudinal oral anticoagulant adherence trajectories in patients with atrial fibrillation. J. Am. Coll. Cardiol. 78 (24), 2395–2404. 10.1016/j.jacc.2021.09.1370 34886959

[B24] Tannous E. E. Selitzky S. Vinker S. Stepensky D. Schwarzberg E. (2025). Predictive modelling of medication adherence in post-myocardial infarction patients: a Bayesian approach using beta-regression. Eur. J. Prev. Cardiol. 32 (8), 649–658. 10.1093/eurjpc/zwae327 39365905

[B25] Whitmore K. Zhou Z. Magnussen C. G. Nelson M. R. Carrington M. J. (2025). Review of strategies to improve adherence to lipid lowering therapy in the primary prevention of cardiovascular disease. Eur. J. Prev. Cardiol., zwaf237. 10.1093/eurjpc/zwaf237 40273404

