# Supplementary material for: Prediction of long-term adherence to direct oral anti-coagulants in patients with atrial fibrillation using first-order Markov models
Source: Front Pharmacol. 2025 Oct 16;16:1673919. doi: 10.3389/fphar.2025.1673919 (PMC12572717; doi:10.3389/fphar.2025.1673919)
Supplement: Supplementary file 1 [file Supplementaryfile1.docx]

**Supplementary material**

1. Figure S1. Attrition diagram


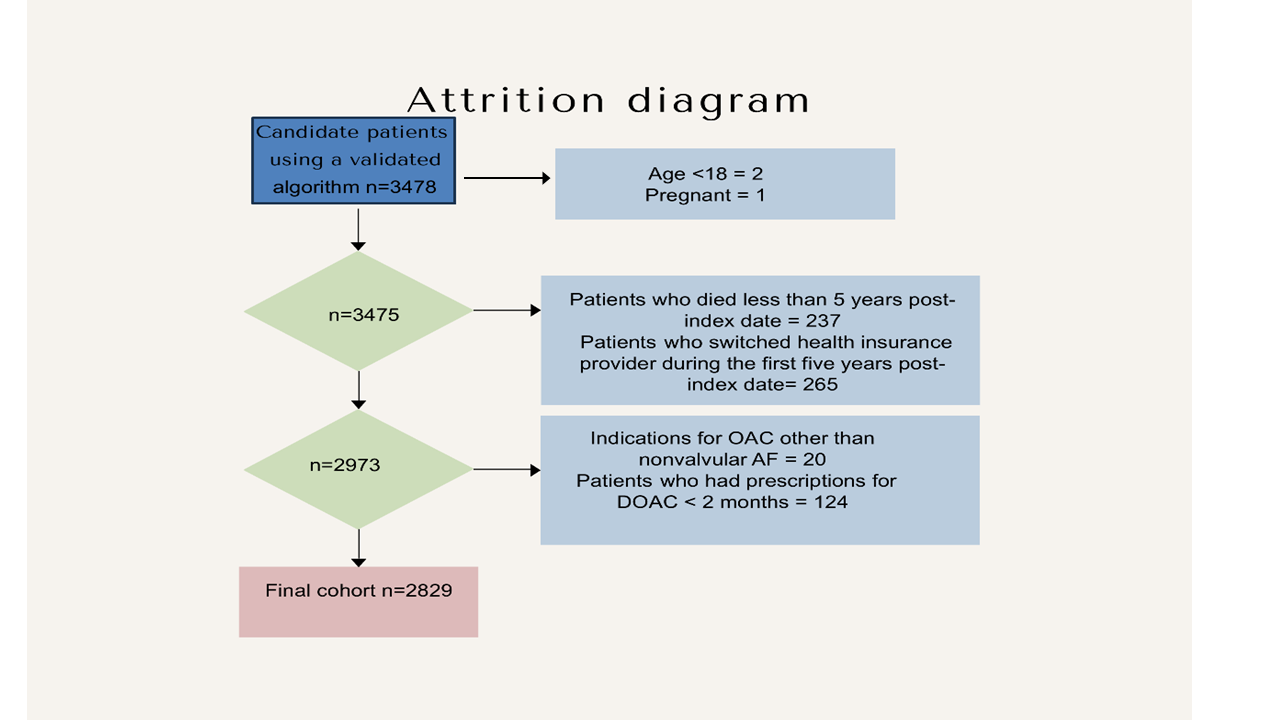


*indications for oral anticoagulants other than nonvalvular AF (ICD-9 code): Pulmonary embolism (415.1), Phlebitis and thrombophlebitis (451) , Other venous embolism and thrombosis (453),Disease of mitral and aortic valve (394,395,395) ,Other rheumatic heart diseases (398)

1. **Sample size calculation**

Since there are no published guidelines for sample size determination for first order Markov proportional odds predictive models, we used the most conservative criterion for sample size determination (the one that will yield the highest minimal required sample size). In this case achieving a global shrinkage factor > 0.95 was chosen.

$$\mathrm{Global}shrinkage= \frac{Model \chi2-df}{Model \chi2}$$

where:

- Model χ² = likelihood ratio chi-square statistic
- df = effective degrees of freedom

We used a simulation-based approach and evaluated several sample sizes (1500,1750,2000, 2250, 2500, 2750,3000) and the model with all the candidate predictive variables (including interactions). Based on these simulations a sample size between 2750-3000 was required to ensure global shrinkage >0.95.

1. **TEN-SPIDERS reporting tool for PDC**

**Table S1: TEN-SPIDERS reporting tool for PDC**

| TEN-SPIDERS reporting tool for PDC | | |
| --- | --- | --- |
| **Threshold** | PDC was analyzed as a continuous variable |  |
| **Eligibility criteria for inclusion in sample** | See the "Methods" section |  |
| **Numerator and denominator** | Denominator = 90 | Numerator: days covered + hospitalization days |
| **Survival** | Patients who died during 5-year follow up were not included |  |
| **Pre-supply** | Not relevant, only new users were included |  |
| **In-hospital supply** | Assumed | Added to numerator |
| **Dosing information** | Available |  |
| **Early refills** | Carry-over was granted for early refills of the same drug |  |
| **Switching** | Carry-over was granted for therapeutic switches | Example: dabigatran 150mg*2 , 60 tablets filled at 01/04/2025 and then switched to Apixaban 5mg*2, filled at 26/4/2025 we assumed that the patient first used all Dabigatran quantity and then started Apixaban |

1. **Additional descriptive statistics for DOAC type and PDC**

**Table S2 Distribution of type of DOAC**

| **DOAC** | **Number of users (%)** |
| --- | --- |
| Apixaban | 1843 (65.1) |
| Dabigatran | 242 (8.6) |
| Rivaroxaban | 413 (14.6) |
| Switched | 331 (11.7) |

**Table S3: Number and proportion of patients at each PDC state across all 90-day windows**

| **PDC** | **Apixaban** | **Dabigatran etexilate** | **Rivaroxaban** | **Switched** | **Overall** |
| --- | --- | --- | --- | --- | --- |
| 0-9% | 5795 (19.7%) | 752 (19.4%) | 1179 (17.8%) | 1249 (23.6%) | 8975 (19.8%) |
| 10-19% | 261 (0.9%) | 47 (1.2%) | 24 (0.4%) | 58 (1.1%) | 390 (0.9%) |
| 20-29% | 302 (1.0%) | 71 (1.8%) | 45 (0.7%) | 81 (1.5%) | 499 (1.1%) |
| 30-39% | 404 (1.4%) | 61 (1.6%) | 56 (0.8%) | 89 (1.7%) | 610 (1.3%) |
| 40-49% | 405 (1.4%) | 62 (1.6%) | 63 (1.0%) | 79 (1.5%) | 609 (1.3%) |
| 50-59% | 448 (1.5%) | 92 (2.4%) | 91 (1.4%) | 92 (1.7%) | 723 (1.6%) |
| 60-69% | 1062 (3.6%) | 168 (4.3%) | 200 (3.0%) | 172 (3.2%) | 1602 (3.5%) |
| 70-79% | 1128 (3.8%) | 192 (5.0%) | 263 (4.0%) | 198 (3.7%) | 1781 (3.9%) |
| 80-89% | 2039 (6.9%) | 309 (8.0%) | 640 (9.7%) | 353 (6.7%) | 3341 (7.4%) |
| 90-100% | 17644 (59.8%) | 2118 (54.7%) | 4047 (61.2%) | 2925 (55.2%) | 26734 (59.1%) |

1. **Sensitivity analysis**

Two additional analyses were performed in which PDC states between 0-9% and 90-100% were collapsed. In the first analysis we fitted the same final model but with four states instead of ten; 0-9% ,10-69%, 70-89%,90-100% and evaluated discrimination ability with state occupancy probability (SOP) distribution width (length of IQR of SOP distribution). (Figure S2)

Figure S2. First sensitivity analysis


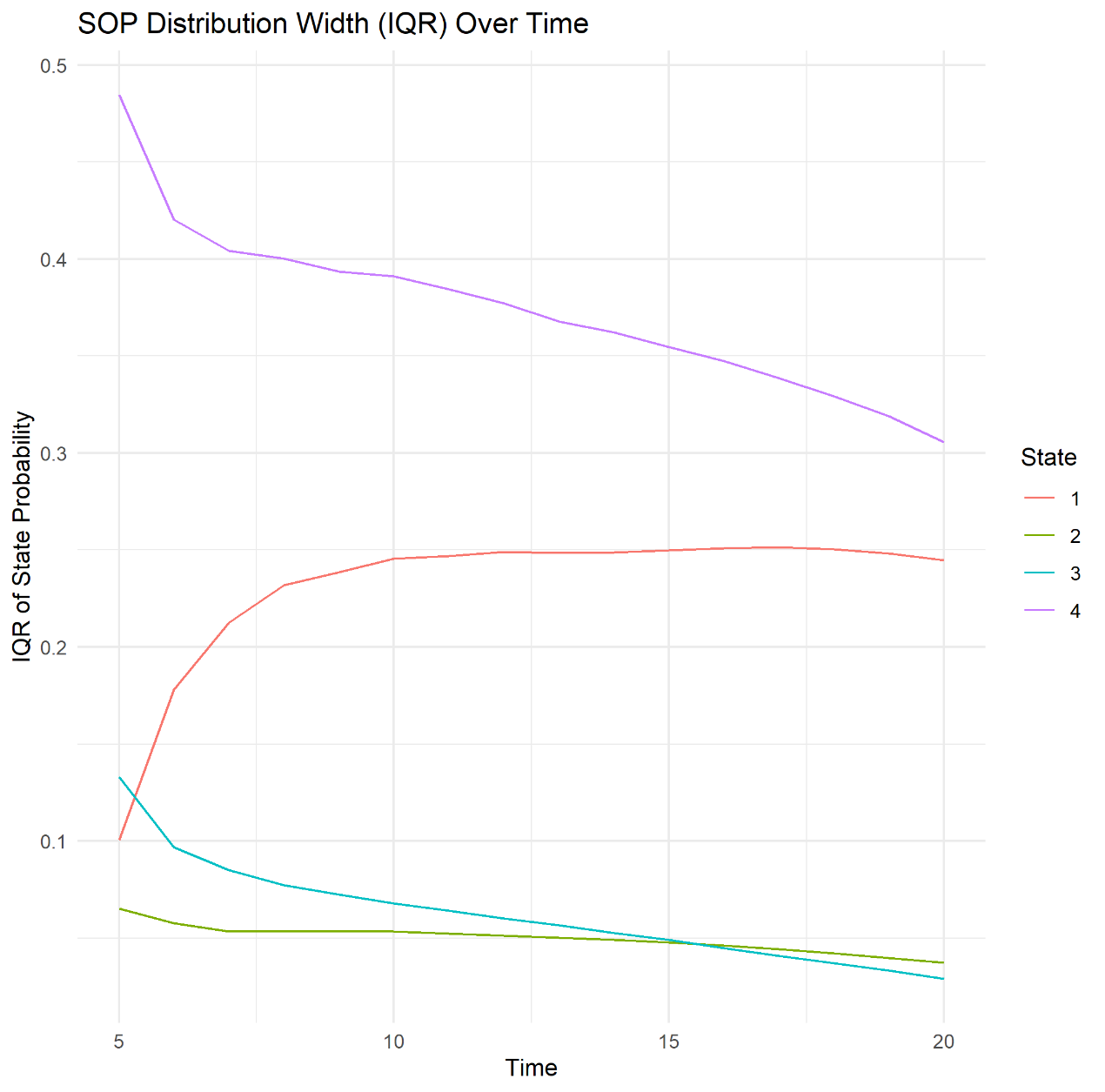


In the second analysis we fitted the same final model but with three states instead of ten; 0-9% ,10-89%,90-100% and evaluated discrimination ability with state occupancy probability (SOP) distribution width (length of IQR of SOP distribution). (Figure S3)

Figure S3. Second sensitivity analysis


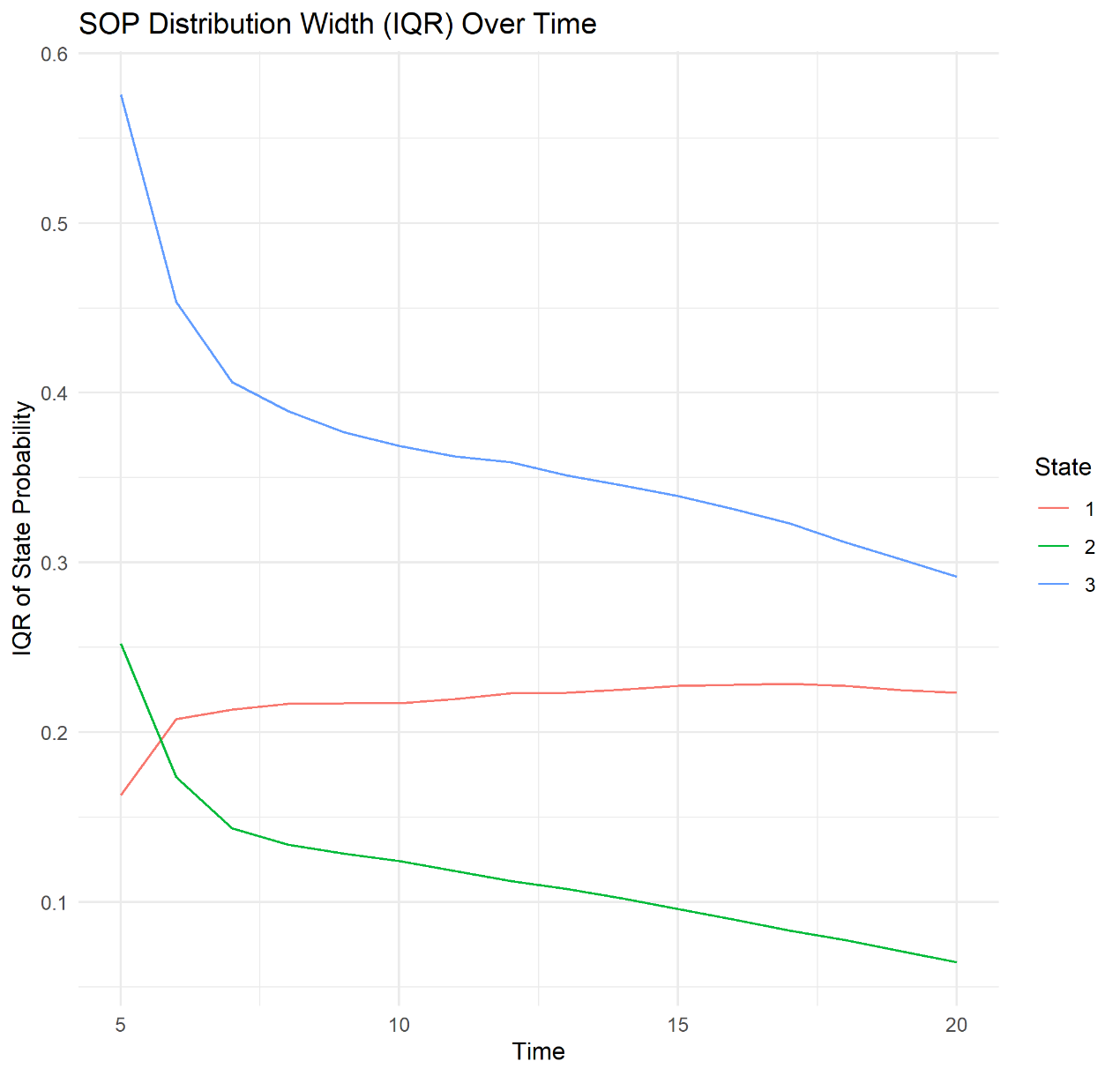


Sensitivity analysis suggests that as in the original analysis discrimination performance is good for the extreme states 0-9% and 90-100% but weak for the intermediate states regardless of the method of grouping.

1. **State transition probabilities calibration plots for PDC states 90-100%, 80-89%, and 0-9%.**

**Figure S4: State transition probabilities calibration plots for PDC state 0-9%.**


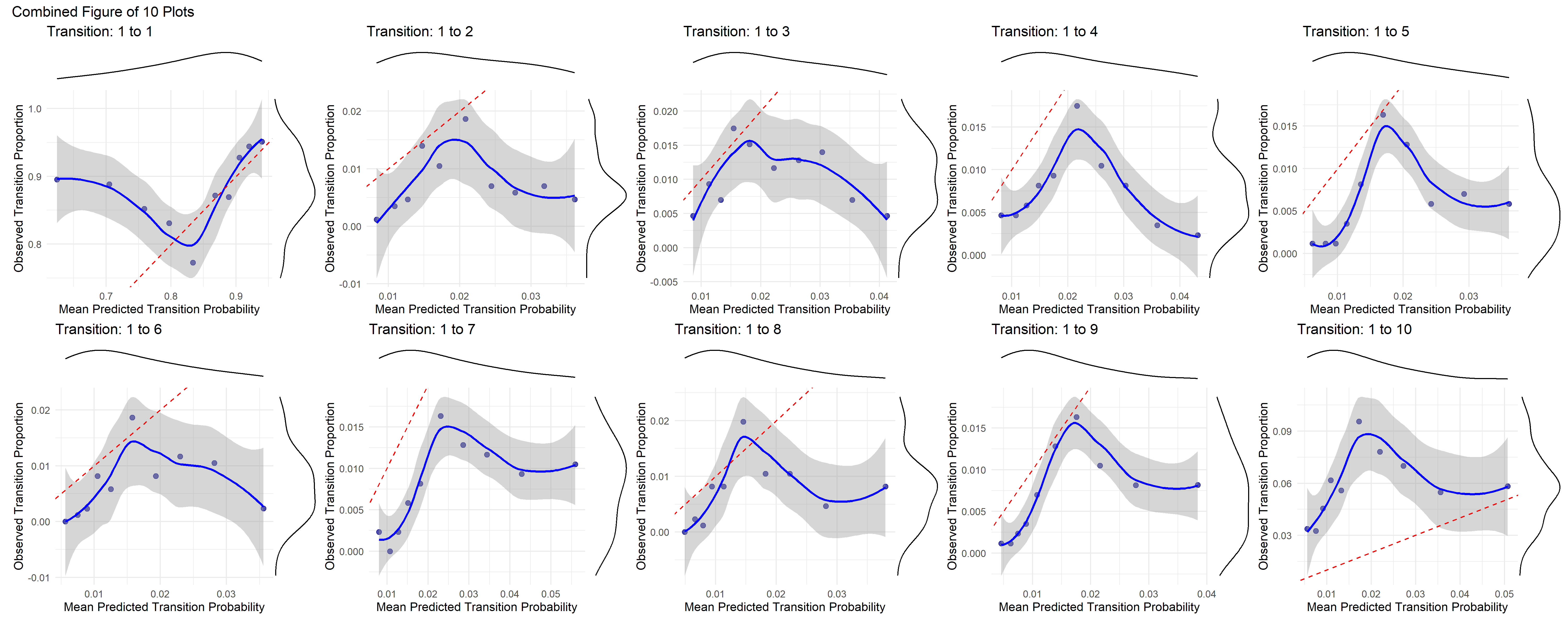


**Figure S5: State transition probabilities calibration plots for PDC state 80-89%.**


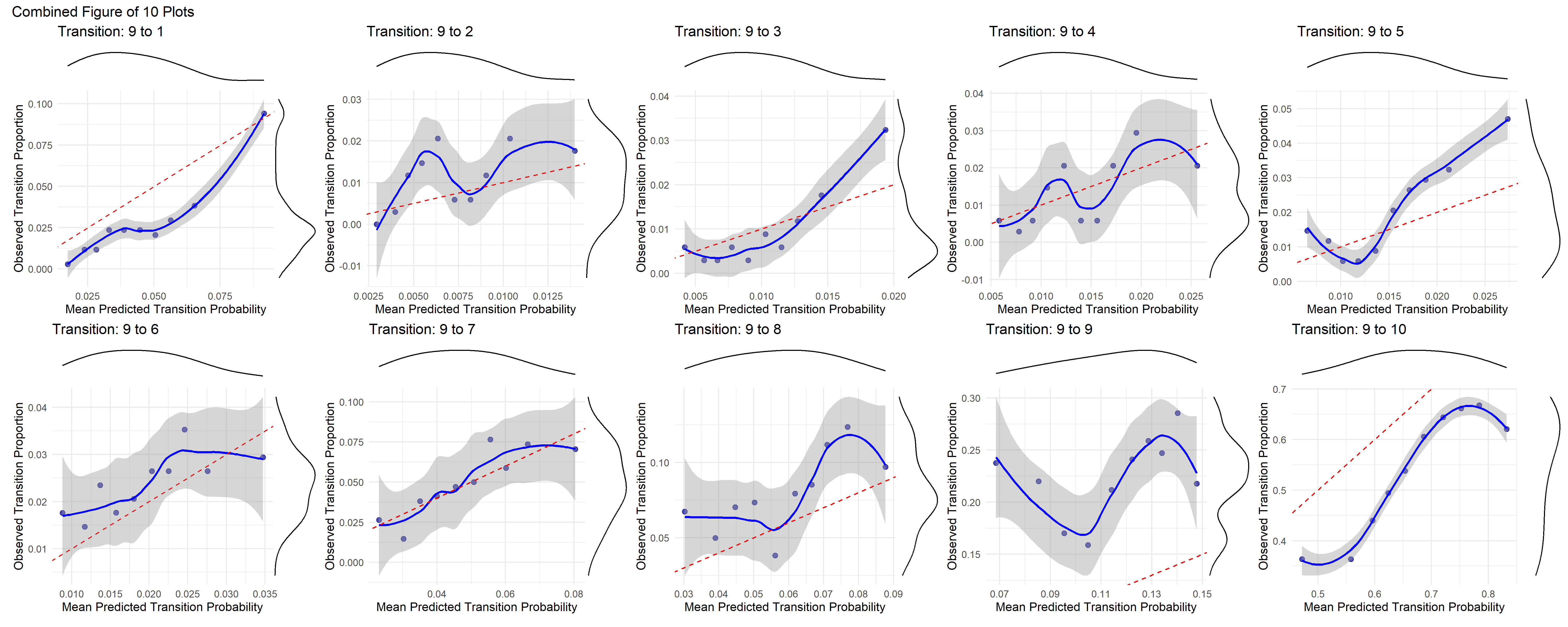


**Figure S6: State transition probabilities calibration plots for PDC state 90-100%.**


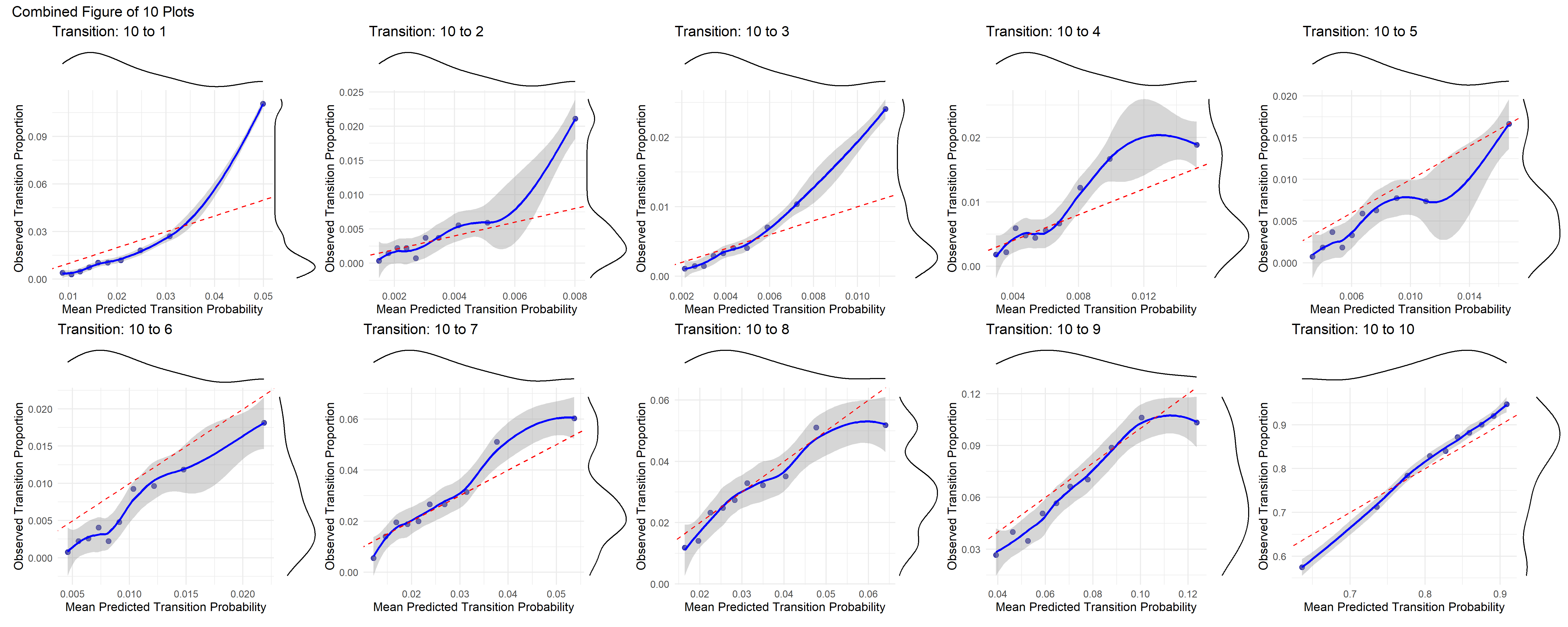


1. **Investigation of possible correlation between Age and CHA2DS2-VASc score**

To check the effect of including CHA2DS2-VASc score on the partial effect of Age we fitted a model with CHA2DS2-VASc score and without CHA2DS2-VASc score. Figure 5 shows partial effects of Age on adherence with and without CHA2DS2-VASc score.

Figure S7 Partial effect of Age on log odds of ordinal PDC category for DOACs


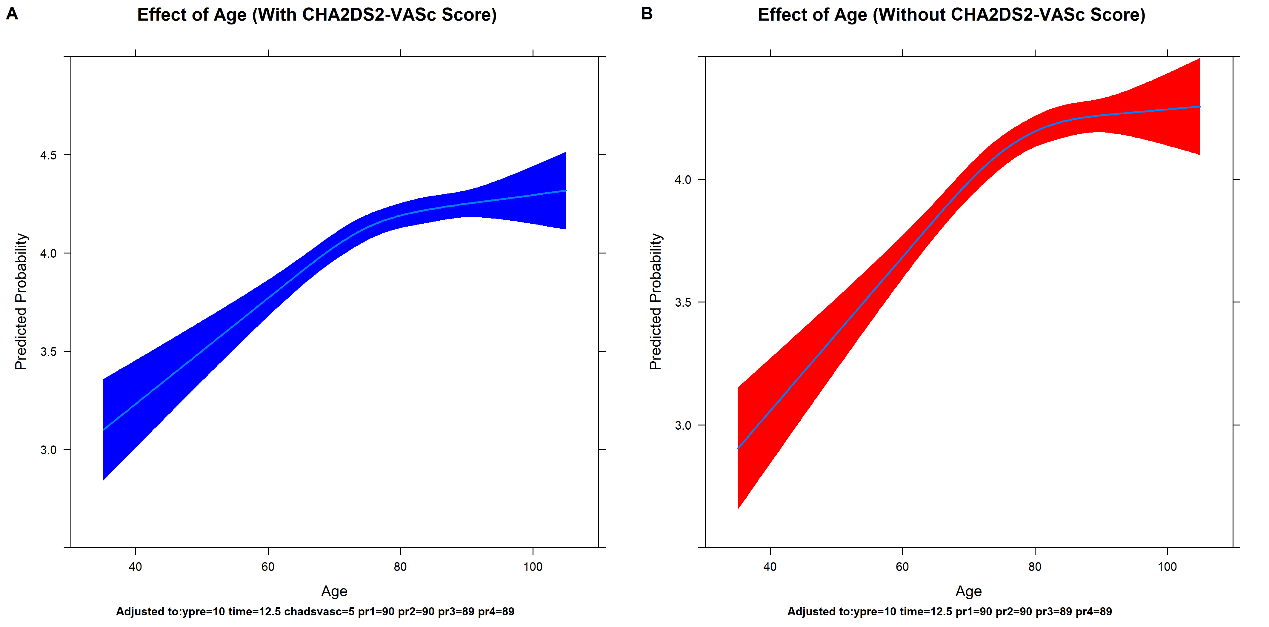


The plateau of the effect of age on adherence remains regardless of CHA2DS2-VASc score. Additionally, we checked linear correlation between Age and CHA₂DS₂-VASc score (although not an optimal test) and it was weak (0.4). Also, we fitted a model of the form: CHA₂DS₂-VASc score ~ rcs(Age,4) and the explained variance was low (Adjusted R-squared = 0.199). Moreover, the effects on the coefficients of Age remain relatively the same in the model with or without CHA2DS2-VASc score:

Model with CHA2DS2-VASc score as predictor:

|  | **y** | | |
| --- | --- | --- | --- |
| *Predictors* | *Odds Ratios* | *CI* | *p* |
| y>=2 | 0.01 | 0.00 – 0.01 | **<0.001** |
| y>=3 | 0.00 | 0.00 – 0.01 | **<0.001** |
| y>=4 | 0.00 | 0.00 – 0.01 | **<0.001** |
| y>=5 | 0.00 | 0.00 – 0.01 | **<0.001** |
| y>=6 | 0.00 | 0.00 – 0.00 | **<0.001** |
| y>=7 | 0.00 | 0.00 – 0.00 | **<0.001** |
| y>=8 | 0.00 | 0.00 – 0.00 | **<0.001** |
| y>=9 | 0.00 | 0.00 – 0.00 | **<0.001** |
| y>=10 | 0.00 | 0.00 – 0.00 | **<0.001** |
| ypre | 1.69 | 1.68 – 1.70 | **<0.001** |
| time | 1.04 | 1.02 – 1.06 | **<0.001** |
| age | 1.03 | 1.02 – 1.04 | **<0.001** |
| age' | 0.98 | 0.96 – 1.00 | **0.031** |
| age'' | 1.05 | 0.96 – 1.14 | 0.305 |
| chadsvasc | 1.04 | 1.03 – 1.06 | **<0.001** |
| pr1 | 1.00 | 0.99 – 1.00 | 0.059 |
| pr1' | 1.00 | 1.00 – 1.01 | 0.287 |
| pr2 | 1.00 | 1.00 – 1.00 | 0.902 |
| pr2' | 1.00 | 1.00 – 1.00 | 0.963 |
| pr2'' | 1.94 | 1.16 – 3.24 | **0.011** |
| pr3 | 1.01 | 1.00 – 1.02 | **0.008** |
| pr3' | 0.99 | 0.98 – 1.00 | 0.076 |
| pr3'' | 61.23 | 14.79 – 253.51 | **<0.001** |
| pr4 | 1.02 | 1.01 – 1.03 | **<0.001** |
| pr4' | 0.99 | 0.98 – 1.00 | 0.079 |
| pr4'' | 124.97 | 22.14 – 705.43 | **<0.001** |
| time * pr3 | 1.00 | 1.00 – 1.00 | 0.098 |
| time * pr3' | 1.00 | 1.00 – 1.00 | 0.126 |
| time * pr3'' | 0.84 | 0.76 – 0.94 | **0.002** |
| time * pr4 | 1.00 | 1.00 – 1.00 | 0.105 |
| time * pr4' | 1.00 | 1.00 – 1.00 | 0.596 |
| time * pr4'' | 0.86 | 0.76 – 0.98 | **0.023** |
| Observations | 45264 | | |
| R^2^ | 0.577 | | |

Model without CHA2DS2-VASc score:

|  | **y** | | |
| --- | --- | --- | --- |
| *Predictors* | *Odds Ratios* | *CI* | *p* |
| y>=2 | 0.00 | 0.00 – 0.01 | **<0.001** |
| y>=3 | 0.00 | 0.00 – 0.01 | **<0.001** |
| y>=4 | 0.00 | 0.00 – 0.01 | **<0.001** |
| y>=5 | 0.00 | 0.00 – 0.00 | **<0.001** |
| y>=6 | 0.00 | 0.00 – 0.00 | **<0.001** |
| y>=7 | 0.00 | 0.00 – 0.00 | **<0.001** |
| y>=8 | 0.00 | 0.00 – 0.00 | **<0.001** |
| y>=9 | 0.00 | 0.00 – 0.00 | **<0.001** |
| y>=10 | 0.00 | 0.00 – 0.00 | **<0.001** |
| ypre | 1.69 | 1.68 – 1.71 | **<0.001** |
| time | 1.04 | 1.02 – 1.06 | **<0.001** |
| age | 1.03 | 1.02 – 1.04 | **<0.001** |
| age' | 0.98 | 0.96 – 1.00 | **0.037** |
| age'' | 1.03 | 0.94 – 1.12 | 0.510 |
| pr1 | 1.00 | 0.99 – 1.00 | **0.048** |
| pr1' | 1.00 | 1.00 – 1.01 | 0.248 |
| pr2 | 1.00 | 1.00 – 1.00 | 0.869 |
| pr2' | 1.00 | 1.00 – 1.00 | 0.912 |
| pr2'' | 1.97 | 1.18 – 3.29 | **0.009** |
| pr3 | 1.01 | 1.00 – 1.02 | **0.008** |
| pr3' | 0.99 | 0.98 – 1.00 | 0.070 |
| pr3'' | 63.69 | 15.38 – 263.73 | **<0.001** |
| pr4 | 1.02 | 1.01 – 1.03 | **<0.001** |
| pr4' | 0.99 | 0.98 – 1.00 | 0.077 |
| pr4'' | 124.63 | 22.07 – 703.75 | **<0.001** |
| time * pr3 | 1.00 | 1.00 – 1.00 | 0.104 |
| time * pr3' | 1.00 | 1.00 – 1.00 | 0.131 |
| time * pr3'' | 0.84 | 0.76 – 0.94 | **0.002** |
| time * pr4 | 1.00 | 1.00 – 1.00 | 0.103 |
| time * pr4' | 1.00 | 1.00 – 1.00 | 0.594 |
| time * pr4'' | 0.86 | 0.76 – 0.98 | **0.024** |
| Observations | 45264 | | |
| R^2^ | 0.577 | | |
